# Analysis of Regulatory T Cell Subsets and Their Expression of Helios and PD-1 in Patients with Hashimoto Thyroiditis

**DOI:** 10.1155/2019/5368473

**Published:** 2019-05-13

**Authors:** Yifang Hu, Lijuan Zhang, Huanhuan Chen, Xiaoyun Liu, Xuqin Zheng, He Shi, Lin Jiang, Dai Cui

**Affiliations:** Department of Endocrinology, Nanjing Medical University First Affiliated Hospital, Nanjing, China

## Abstract

Hashimoto thyroiditis (HT) is an autoimmune disease that presumably arises consequent to loss of immune tolerance to autoantigen in thyroid. Regulatory T cells (Tregs) are considered to play a vital role in maintaining the immune balance, as they own intensive suppressive function. This study was undertaken to analyze numbers of Tregs and their expressions of Helios and PD-1 in HT patients. It also aimed to explore the relationship of these with thyroid function and specific autoantibodies. Peripheral blood mononuclear cells (PBMCs) were extracted from blood of 20 healthy controls (HC) and 42 HT patients with varying thyroid functions (10 overt hypothyroidism, 12 subclinical hypothyroidism, and 20 euthyroidism). We performed flow cytometry analysis in PBMCs to detect CD4^+^CD25^+^Foxp3^+^Tregs and their subsets, including CD45RO^+^Foxp3^high^ activated Treg cells (aTregs), CD45RO^−^Foxp3^low^ resting Tregs cells (rTregs), and CD45RO^+^Foxp3^low^ secreting Treg cells (sTregs), as well as the expression of Helios and PD-1 on these cells. The results showed that the percentage of Tregs, aTregs was significantly lower in HT patients and it showed inverse correlation to thyroid function states, in comparison with these in healthy controls. In addition, patients with HT showed decreased expression of Helios in aTregs, while having increased expression of PD-1 in Tregs and sTregs. The levels of Tregs, aTregs, and Helios expressing aTregs were all negatively correlated with antithyroid antibodies. In conclusion, the deficiency of Tregs frequency and aberrant expressions of Helios and PD-1 may possibly contribute to thyroid immune damage in HT.

## 1. Introduction

Hashimoto thyroiditis (HT), the most common autoimmune thyroid disease (AITD), is an organ-specific immune disease characterized by immune T cells and antibody-mediated process [1]. It is suggested that genetic and environmental factors result in the loss of self-tolerance to thyroid antigens and CD4^+^T cell activation, which triggers overflowing immune responses, and eventually induce lymphocytic infiltration within thyroid and increase serum antithyroperoxidase antibody (TPOAb) and antithyroglobulin antibody (TGAb) [2]. Meanwhile, there is a crowd of cells with immunosuppressive function. Among these, regulatory T cells (Tregs) play a crucial role in maintaining homeostasis by dampening and preventing the excessive inflammatory reactions. Previous studies have exposed the evidence that the developmental or functional anomalies of Tregs are tightly correlated with Hashimoto thyroiditis [3–5].

In recent years, advances in our cognition of Tregs have resulted in the discovery of various subtypes, including mainly IL-10-producing Tr1 (type 1 Treg), TGF-*β*-producing CD4^+^ Th3 (T helper 3), and CD4^+^CD25^+^ Foxp3 Tregs. Among these, CD4^+^CD25^+^Foxp3^+^ T cells have been a typical and recognized characterization of Tregs up to now. Foxp3 is a master transcription molecule and functional regulator of Tregs, which plays a crucial role in conferring suppressive function to CD4^+^CD25^+^ Tregs by amplifying the expression of Treg cell-type gene such as CD25, IL-10, and CTLA-4 [6]. In addition, there is a couple of specific surface marker CD45RO/CD45RA, which is coded by a single gene on chromosome 1 (1q32), contributing to the development and progression of CD4^+^ lymphocytes. According to the division of different phenotypes, T lymphocytes are classified into naive T cell (CD45RA+ or CD45RO- T cells) and induced T cells (CD45RA- or CD45RO+ T cells), which are formed in peripheral blood by cytokines stimulation [7]. Recent studies [8, 9] have shown that the combinatorial expression of Foxp3 and CD45RO can separate CD4^+^CD25^+^ Tregs into three functionally and phenotypically distinct subpopulations, consisting of CD45RO^+^Foxp3^high^ activated regulatory T cells (aTregs), CD45RO^−^Foxp3^low^ resting regulatory T cells (rTregs), and CD45RO^+^Foxp3^low^ cytokine-secreting regulatory T cells (sTregs). The former two subpopulations confer suppressive function, and aTregs possess highly suppressive ability in vivo. However, sTregs are nonsuppressive and can secrete large amounts of cytokines, such as IL-17, IL-2, and IFN-*γ* [10, 11]. Meanwhile, relying on CD45RO expression, CD4^+^CD25^−^ effector T cells can be divided into CD45RO^−^ naive T_effect_ and CD45RO^+^ memory T_effect_ cells. The later one could respond to antigen and display immediate effector function.

Although almost all animal experiments [3, 12–14] have revealed that the absence of Tregs may result in spontaneous progress of autoimmune thyroiditis, the investigation of Tregs in HT patients is not as unambiguous. Some clinical evidence [15, 16] has supported the decreased frequency of circulating Tregs in human subjects with HT, but several conflicting findings were also reported. For example, Bossowski et al. [5] observed lower proportions of CD4^+^CD25^high^ and CD4^+^FoxP3^+^, but there were no significant reductions of CD4^+^CD25^+^CD127^low^Foxp3^+^ and CD4^+^CD25^int^ Tregs in children with AITD. Marazuela et al. [17] suggested that the levels of CD69^+^NKG2D^+^IL-10^+^ Tregs were increased in peripheral blood from AITD adults. With regard to these results, the different detection markers or subsets with distinct phenotypes may attribute to such discrepancy. Therefore, we mainly focus on investigating the most physiologically relevant CD4^+^CD25^+^Foxp3^+^ Tregs in patients with newly diagnosed HT. Given the dissection of Tregs into subpopulations and little data reported in autoimmune thyroiditis, we sought to identify the proportional change of Tregs subpopulations in normal and HT states.

Beyond estimating Tregs number, research on functional analysis of Tregs in AITD is limited and inconsistent. It has been indicated that IL-10, IL-2, IL-35, and TGF-*β* could be critical cytokines for Tregs activities [18–20]. Of note, two recent markers Helios and PD-1 may also exert vital function in regulating Treg cell peripheral tolerance and autoimmunity. Helios, a member of the Ikaros family, is regarded as important mediator for Tregs since it can upregulate Foxp3 expression by attaching to the Foxp3 promoter [21, 22]. It has been acknowledged that Tregs coexpressing Foxp3 and Helios possess better suppressive abilities than CD4^+^CD25^+^ Tregs [23]. To the best of our knowledge, there has not been any research on Foxp3^+^Helios^+^ Tregs in the field of thyroid reported. PD-1 belongs to B7 family and delivers inhibitory signal to prevent immune damage when binding to its ligands PD-L1. In human, the role of PD-1/PD-L1 pathway has been observed in various autoimmune diseases such as rheumatoid arthritis (RA), systemic lupus erythematosus (SLE), multiple sclerosis (MS), and Type 1 diabetes mellitus (T1DM) [24], while little is known in autoimmune thyroiditis. Given that, studies exploring the possible associations of PD-1 and Helios expressing Tregs with HT are warranted.

In this study, we attempted to perform a quantitative and functional analysis of CD4^+^CD25^+^ Tregs and its diverse subsets in HT patients due to inconsistent results and limited reports. Considering that HT would suffer different extent immune damage based on frequency of antibodies and then experience three clinical stages defined by thyroid function (euthyroidism, subclinical hypothyroidism, and overt hypothyroidism), Tregs changes and correlations with thyroid autoantibodies are necessary to be recognized in depth as well.

## 2. Materials and Methods

### 2.1. Subjects

HT patients were recruited from the Endocrinology Outpatient Department of the First Affiliated Hospital of Nanjing Medical University. A total of 42 patients with HT were enrolled in the study according to the following criteria: (1) newly diagnosed HT patients who had never been treated with thyroid hormone replacement or any immune regulators. (2) Laboratory examination showed a normal or increased thyroid-stimulating hormone (TSH) level with elevated antibodies, TPOAb (> 34 IU/mL) or/and TGAb (> 115 IU/mL). (3) Ultrasonography (US) demonstrated a diffuse, enlarged, and heterogeneous thyroid. Patients were classified into three subgroups on the basis of thyroid hormone levels, comprising euthyroidism (eHT), subclinical hypothyroidism (sHT), and overt hypothyroidism (oHT). The laboratory diagnosis of eHT was HT patients with normal levels of FT3, FT4, and TSH; sHT group was HT patients with elevation in levels TSH along with normal levels of FT3 and FT4; oHT group was HT patients with deficient levels of FT4 and raised levels of TSH. During the same period, age- and sex-matched 20 healthy euthyroid volunteers with negative antithyroid antibodies and no history of AITD were recruited as healthy controls (HC).

Exclusion criteria for all subjects were as follows: (1) presence of other autoimmune diseases, such as SLE, RA, MS, or T1DM, (2) acute infections or septicemia, (3) severe systemic disease or chronic disease, (4) current pregnancy and lactation, and (5) the use of anti-inflammatories or immunomodulators.

The study was approved by the Ethics Committee of Nanjing Medical University First Affiliated Hospital (2017-SR-141). Informed consents were obtained from all subjects included in the study.

### 2.2. Blood Samples

Peripheral venous blood (3ml) was drawn from the enrolled subjects after signing the informed consent. Fresh blood samples collected in vacutainer tubes (green head) containing 0.2 ml heparin were prepared for peripheral blood mononuclear cells (PBMCs).

### 2.3. Flow Cytometric Analysis

*(1) Sample Preparation*. PBMCs were aseptically isolated using Ficoll-Hypaque density centrifugation within 2 hours of blood being drawn. In brief, blood samples were diluted 1:1 with RPMI-1640 (Gibco, Grand Island, USA) and slowly dropped in the inclined tubes with lymphoprep, then centrifuged for 30 min at 800 g, 20°C. Mononuclear cells acquired from the middle gradient interface were washed twice and suspended in phosphate buffered saline (PBS).

*(2) Cell Surface Staining.* Nearly 1 × 10^6^ PBMCs were stained and incubated for 30 min at 20°C in darkness with 3 *μ*l (according to optimisation assays) of following monoclonal antibodies: anti-CD3 APC-H7 (BD Bioscience, New Jersey, USA), anti-CD4 BV510 (BD Bioscience, New Jersey, USA), anti-CD25 PE-cy7 (BD Bioscience, New Jersey, USA), anti-CD45RO BV421 (BD Bioscience, New Jersey, USA), and anti-PD-1 PE (BioLegend, Santiago, USA). Then, cells were washed twice in 2 ml PBS at 3000 r 4°C for 5 min and remove the supernatants.

*(3) Intracellular Cytokine Staining.* After fixation and permeabilization with Fix/Perm kit (eBioscience, San Diego, CA, USA) at 4°C for 45 min, cells were stained intracellularly with 4 *μ*l of anti-Foxp3 Alexa Fluor 647 and anti-Helios FITC (BioLegend, Santiago, USA) at 20°C for 30 min in darkness. Then, cells were washed twice in 2 ml perm/wash buffer (1×) and centrifuged 5 min at 3000 r 4°C. All the steps of the experiment were strictly performed according to the manufacturer's instructions.

*(4) Multicolor Flow Cytometric Analysis.* The stained cells were identified, quantified, and classified on seven-color flow cytometry (FCM, FACS Verse, BD Biosciences, USA) and Flow Jo 10. Lymphocytes were first gated, followed by CD4+ Th cells based on CD3 and CD4 expression. Quadrants of Tregs were set according to the expression of CD25 and Foxp3 on CD4+ cells. The CD4+ cells without CD25 and Foxp3 expression were defined as Teffect cells. The three Treg subsets were differentiated by the levels of CD45RO and Foxp3, including Foxp3^hi^CD45RO^+^ aTregs, CD45RO^+^Foxp3^lo^ sTregs, and CD45RO^−^Foxp3^lo^ rTregs. Similarly, Teffect cells could be separated into memory CD45RO^+^ Teffect and initial CD45RO^−^ Teffect cells. Based upon above phenotypes, we further analyzed Helios ^+^ and PD-1 levels in Treg, Teffect cells and their subsets.

### 2.4. Statistical Analysis

All statistical data were calculated and analyzed by SPSS 25 (IBM SPSS Software, Inc., Chicago, USA) and GraphPad Prism 5.0 software (GraphPad Software, Inc., San Diego, CA, USA). Continuous data were represented as mean ± standard deviation (SD) or median with percentiles (25-75 percentiles) on the basis of the distribution, which was detected by Shapiro-Wilk normality test. For normally distributed data, comparisons between two groups were analyzed by independent-sample T test, while more groups by one-way ANOVA and multiple comparison test (LSD, Tukey). For abnormal distribution series, we used the nonparametric tests to examine the differences within groups or between groups. Specifically, the Kruskal–Wallis test was used in comparisons among three or more groups, Wilcoxon-Mann-Whitney test was utilized to test the heterogeneity between two groups, and unpaired/Welch's correction was applied for samples with unequal variances. Pairwise correlations between different markers were performed by Spearman's correlation test.* P *values < 0.05 were considered to be statistically significant.

## 3. Results

### 3.1. Characteristics of the Subjects

Clinical and demographic features of patients with HT and HC were shown in Table 1. Samples derived from 20 HC, 20 eHT, 12 sHT, and 10 oHT were included in our study. There was a similar age distribution among all groups. Most of the participants were females, which was consistent with the epidemiological characteristic of HT. When compared to HC group, the FT3 and FT4 levels were significantly lower (*P = *001,* P *< 0.0001, respectively) in oHT group, while TSH was higher in both sHT and oHT group (*P *< 0.0001,* P =* 0.002, respectively). A statistically significant increase in TPOAb and TgAb was observed in all subgroups of HT patients, but having a marked increase in overt hypothyroidism (*P *< 0.0001).

### 3.2. The Percentage of Treg Cells

**Figure 1:**
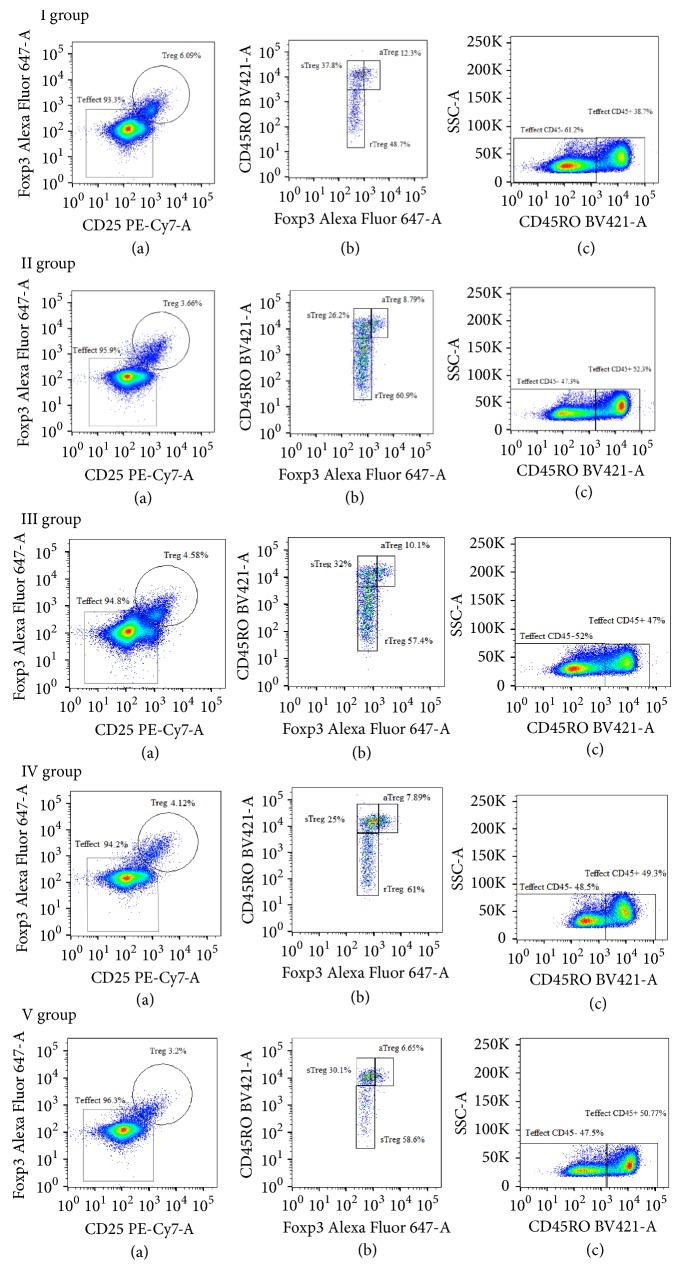
*Representative flow cytometric dot plots of the T cell subsets in peripheral blood of subjects. I group represent the healthy controls, II group represent HT patients, III group represent euthyroidism HT, IV group represent subclinical hypothyroidism HT, and V group represent overt hypothyroidism HT.* (a) Gating Treg cells (CD25^+^Foxp3^+^/CD3^+^CD4^+^T cells), the remainder was regarded as Teffect cells. (b) Treg cells were classified into three subsets, aTreg (CD45RO^+^Foxp3^hi^), sTreg (CD45RO^+^Foxp3^lo^), and rTreg (CD45RO^—^Foxp3^lo^). (c) Teffect cells were divided into two subsets, CD45RO^+^Teffect and CD45RO^--^Teffect.

**Figure 2:**
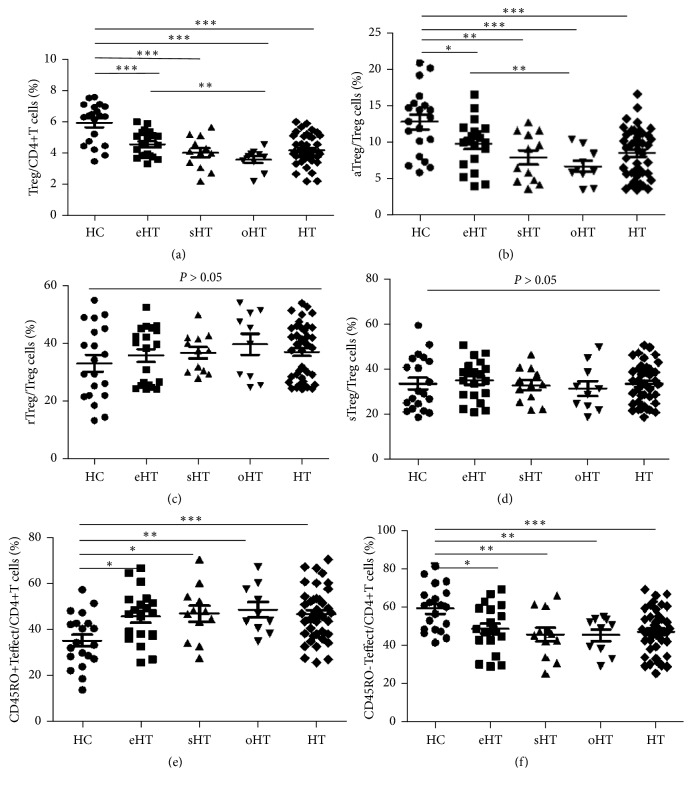
*Differences of T lymphocyte subpopulations in HT patients and healthy controls.* Percentages of (a) Treg in CD4^+^T cell, (b) aTreg in Treg cells, (c) rTreg in Treg cells, (d) sTreg in Treg cells, (e) CD45RO^+^Teffect in CD4^+^T cells, and (f) CD45RO^--^Teffect in CD4^+^T cells (*∗*P < 0.05, *∗∗*P < 0.0075, *∗∗∗*P < 0.0001).

Compared to HC, the analysis of Treg cells in patients with HT revealed a statistically significant decrease in the percentage of Treg cells (4.19 ± 0.93% vs. 5.92 ± 1.27%,* P *< 0.0001, Figure 2(a)), but no difference in Teffect cells. To further explore the differences, we performed a multiple analysis of different subsets in the stage of HT progress. The aTregs in HT and all subgroups were significantly lower than in HC (HT vs. HC, 8.5 ± 3.35% vs.12.75 ± 4.52%,* P *< 0.0001, Figure 2(b)), while neither rTregs (*P *>0.05, Figure 2(c)) nor sTregs (*P *>0.05, Figure 2(d)) were different between groups. In HT patients, Treg cells, as well as aTregs, seemed to trend downward between eHT, sHT, and oHT subgroups; there was a significant difference between eHT and oHT patients. Additional analysis of Teffect subgroups in HT patients showed that the proportion of CD45RO^+^ Teffect in CD4^+^T cells was significantly enhanced (46.65 ± 11.11 vs. 35.25 ± 11.47,* P *< 0.0001, Figure 2(e)), but conversely CD45RO^−^Teffect was reduced compared to HC (46.96 ± 11.43 vs. 58.98 ± 11.58,* P *< 0.0001, Figure 2(f)). The representative flow cytometric dot plots and analysis results are shown in Figures 1 and 2.

### 3.3. Helios Expression in Treg Cells

**Figure 3:**
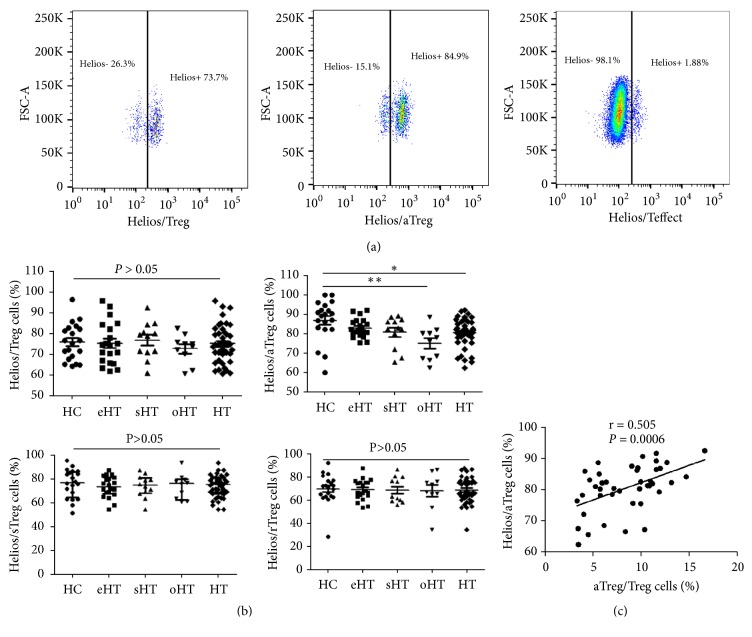
*Helios expression in Treg cells.* (a) Representative flow cytometric plots of Helios staining in Treg, aTreg, and Teffect cells from one HT patient. (b) Comparison of Helios expressing Tregs and subsets between different study groups. (c) Correlation between Helios expression and aTreg cells from HT patients (*∗*P < 0.05; *∗∗*P < 0.0075; *∗∗∗*P < 0.0001).

As shown in Figure 3(a), a high level of Helios was expressed in Tregs, followed by a rather higher expression in aTregs in contrast to Teffect cells with diminished Helios expression. We focused on investigating the proportion of Tregs and subsets expression of Helios between HT patients and healthy controls. There was no significant differences of Helios expression in circulating total Tregs among different groups (*P *>0.05, Figure 3(b)). For Tregs subsets, aTregs exhibited profoundly reduced expression of Helios in HT patients (*P *= 0.019, Figure 3(b)) as compared with HC, and the difference was more obvious in hypothyroid patients (*P *= 0.0028, Figure 3(b)). Moreover, we observed the expression of Helios had a positive correlation with the percentage of aTregs in HT patients (r_s_ = 0.505,* P* = 0.0006, Figure 3(c)). However, sTregs and rTregs expressing Helios showed no significant differences among all groups (*P *>0.05, Figure 3(b)).

### 3.4. PD-1 Expression in Treg Cells

**Figure 4:**
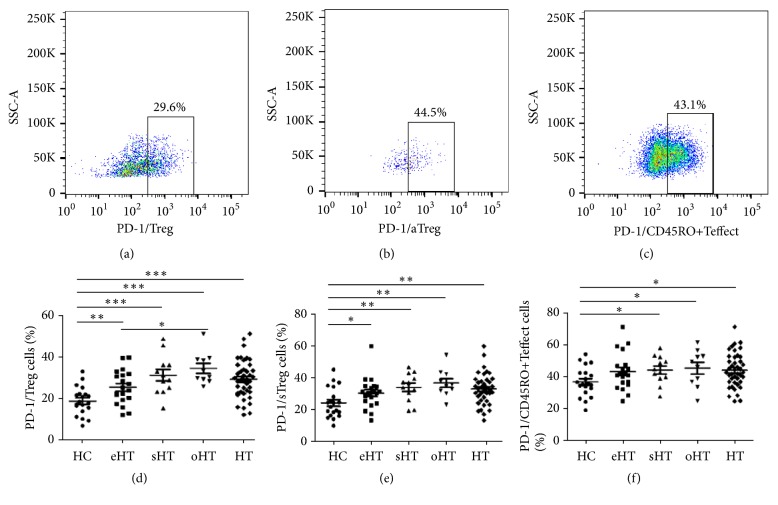
*PD-1 expression on Treg and Teffect cells.* Representative flow cytometric plots of PD-1 staining in (a) Treg, (b) aTreg, and (c) CD45RO^+^Teffect cell from one HT patient. Difference of the percentage of PD-1 within (d) Treg cells, (e) sTreg cells, and (f) CD45RO^+^ Teffect cells among different groups (*∗*P < 0.05; *∗∗*P < 0.0075; *∗∗∗*P < 0.0001).

We analyzed PD-1 expression in Treg and Teffect cells derived from HC and HT patients, as shown in Figure 4. Similar to previous studies, PD-1 was upregulated on aTregs, sTreg, and CD45RO^+^ Teffect cells, while being lowly expressed on rTregs and CD45RO^−^ Teffect cells in both groups. Importantly, compared to HC, expressions of PD-1 on Tregs, sTregs, and CD45RO^+^ Teffect cells were significantly higher in HT patients and subgroups. However, there were no significant differences of PD-1 expression on rTregs, aTregs, or CD45RO^−^Teffect cells between groups.

### 3.5. Correlation Analysis

**Figure 5:**
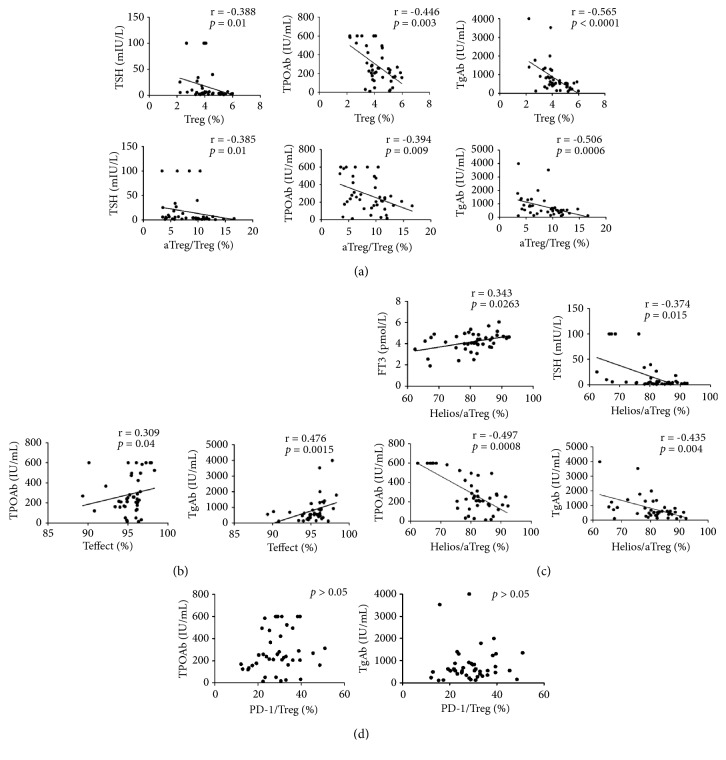
*Spearman pairwise correlation analysis in HT patients between* (a) the Treg or aTreg ratio and TSH, TPOAb or TgAb levels. (b) The Teffect ratio and TPOAb or TgAb levels. (c) The proportion of Helios in aTreg and FT3, TSH, TPOAb or TgAb levels. (d) Proportion of PD-1 in Treg and TPOAb or TgAb levels (r: Spearman correlation coefficient, significance P < 0.05).

To examine whether there was any correlation between the percentage of Tregs, Helios or PD-1 expression and clinical characteristics of HT patients, we performed Spearman's correlation analysis on pooled samples. A series of significant relationships were observed. The percentage of Tregs correlated inversely with serum levels of TSH (r = -0.388,* p = *0.01), TPOAb (r = -0.446,* p = *0.003), and TgAb (r = -0.565,* p *< 0.0001), as did the classic Treg subset, aTregs (r = -0.385,* P = *0.01; r = -0.394,* P = *0.009; r = -0.506,* P *= 0.0006, respectively), as shown in Figure 5(a). Additionally, we found a significant positive correlation between Teffect ratio and TPOAb (r = 0.309,* P =* 0.04) or TgAb (r = 0.476,* P = *0.0015), as shown in Figure 5(b). Moreover, the proportion of aTreg expressing Helios showed a positive correlation with serum FT3 levels (r = 0.343,* P =* 0.0263), whereas inverse correlation with serum TSH concentrations (r = -0.374,* P = *0.015), and TPOAb (r = -0.497,* P = *0.0008) or TgAb titers (r = -0.435,* P = *0.004), as shown in Figure 5(c). However, there were no significant associations between PD-1 status and clinical parameters in HT, as shown in Figure 5(d).

## 4. Discussion

The pathogenesis of HT so far has not been completely explored, although HT is widely considered to be a Th1-mediated autoimmune disease. Various studies have demonstrated that Tregs play an important role in preventing autoimmunity and restraining responses of effector T cells, but the frequency and function of Tregs in the progression of AITD are discrepant [4, 25, 26]. Defective Tregs frequency has been widely reported to exist in Graves' disease [27–32], while less evidence is available regarding Tregs in HT. In this regard, we assessed the classical CD4^+^CD25^+^Foxp3^+^ Tregs number and two key functional indicators in HT patients and HC.

In this study, we found that the percentage of Tregs was apparently reduced in PBMC of HT patients compared to controls. Previously, significant decreases in the number of CD4^+^Foxp3^+^ Tregs, CD4^+^CD25^high^ Tregs, CD4^+^CD25^+^CD127^low^ Tregs, and CD4^+^CD25^high^Foxp3^+^ Tregs were subsequently reported in peripheral blood of HT patients [5, 16, 33]. Our result confirmed the observations from these studies, but some conflicting findings were discovered in other studies. Glick et al. reported that no differences existed in the frequency of CD4^+^CD25^high^ Tregs between AITD, HT, GD, and HC [4]. Marazuela et al. detected a significantly higher proportion of CD4^+^Foxp3^+^ Tregs in AITD patients [25]. The discrepancy may be attributed to differences in surface markers, gating strategies of FCM or analysis methods. We investigated the classical CD4^+^CD25^+^Foxp3^+^ Treg phenotype which was widely used in autoimmune diseases, but also supplied novel evidence of Treg subpopulations in different clinical stages of HT and their associations with clinical features.

Treg cells were delineated as three subpopulations: CD45RO^+^Foxp3^high^ aTregs, CD45RO^−^Foxp3^low^ rTregs, and CD45RO^+^Foxp3^low^ sTregs. Among them, aTreg is a critical suppressor that inhibits responder cells from proliferating and secreting cytokines. The current study showed that aTregs were significantly decreased in HT patients of the variable disease states accompanying decrement of total Tregs. However, there were no significant changes in the proportion of rTreg or sTreg between HT patients and HC. It has previously been reported that the decrement of aTreg not rTreg contributes to the decline of total Tregs in rheumatoid arthritis (RA) and Behcet's disease (BD) [34]. These facts indicate that the proportional reduction of activated Tregs may participate in the pathogenesis of HT. In addition, we observed that Tregs and aTregs stepwise decreased during the clinical stages of HT and negatively correlated with TSH, TPOAb, or TgAb levels. Presumably, the dynamics of aTregs would appear to reflect the severity and activity of disease. As described in systemic sclerosis (SSc), it showed a gradual decrease of the circulating aTreg subset with the progression of disease [35]. Moreover, Miyara et al. demonstrated that rTregs could be converted into aTregs in vivo, and in active SLE, there was a proportional decrease of aTregs, while having an increase of rTregs [8]. Regarding our study, aTregs decreased gradually along with the increase of TSH, TPOAb, TGAb levels. It was implied that HT disease severity and thyroid dysfunction became more apparent with the decreased aTreg cells. Supporting these findings, it was also revealed in HT patients that the proportion of CD45RO^+^ memory effector T cells was increased and positively correlated with TPOAb and TgAb, which was compatible with impairment of immunosuppressive Tregs.

Apart from this, we also assessed the critical function of Tregs in HT patients. Helios is a novel marker and upregulated in Tregs, conferring stable phenotype and stronger immunosuppressive function. It was recently shown that Foxp3^+^Helios^+^Tregs had a greater suppressive ability than either Foxp3 or Helios expressing Tregs, and there was a positive correlation between the two molecules [36]. In our study, aTregs possessed a relatively high expression of Helios, which indicates aTregs may have stronger immunosuppression. However, we found the expression of Helios in aTreg subset was significantly lower in HT patients than in HC. Besides, Helios expressing aTreg was negatively correlated with serum TSH, TPOAb, or TgAb levels. These findings of Helios were in line with those of aTreg in HT patients. It is probably due to the fact that Helios is an intracellular marker, directly attaching to the Foxp3 promoter, thus controlling the suppressive capabilities of Tregs. With respect to the consequence of Helios, we speculate that the reduction of Helios expression preludes the development of HT. However, outcomes of Helios in autoimmune diseases may differ according to the dependency of each disease on Tregs function. Preceding studies reported the increased expression of Helios in RA [22], as well as high levels of Helios^+^ Tregs in SLE [37]. In this paper, the role of Helios in HT was studied for the first time, but the exact mechanism remains not well understood. Hereafter, more extensive researches are still needed to explicate in detail the functional importance of Helios in the progression of HT.

PD-1 is considered as another functional biomarker for CD4^+^ T cells. It is preferentially expressed on activated Treg or Teffect cells. The interaction of PD-1 and PD-L1 can maintain homeostasis between protective responses of Treg and hyperactivated immunoreaction of Teffect cells [38]. The majority of the studies indicated that blocking PD-1/PD-L1 pathway masked Treg mediated inhibition of effector T cells, thus supporting PD-1-dependent Treg suppressive function [39–42]. Contrary to these findings, Franceschini et al. argued that PD-L1 blockade resulted in expansion of Tregs by restricting STAT-5 phosphorylation, suggesting PD-1/PD-L1 contraregulated Treg function [43]. It was tricky to understand the opposite outcome, perhaps the mechanism of PD-1/PD-L1 pathway was influenced by phenotypic diversity of Tregs or microenvironment of diseases. So far in human autoimmune diseases, there has been reported that the level of PD-1 expression on Tregs is significantly lower in SLE, while higher in active generalized vitiligo (GV) patients compared to the controls [44, 45]. However, the role of PD-1 has not been studied in thyroiditis. Here, we first proposed that HT patients showed increased PD-1 expression on Treg and Teffect cells, especially on CD45RO^+^Foxp3^low^ sTreg and CD45RO^+^ Teffect subsets. Classically, HT is regarded to be a Th1-mediated disease, caused by strong inflammatory cytokines infiltration. The CD45RO^+^ Teffect cells are memory effector cells that produce IL-2, IFN-*γ* or IL-4 and associated with the pathogenesis of HT. Besides, sTregs are considered as nonregulatory T cells since they have no suppressive properties and emulate Teffect function. Given that PD-1 expresses inhibitory signals, it may be hypothesized that increased PD-1 expression on sTreg and Teffect cells suppressed the proliferation of autoreactive T cells in a negative feedback manner. However, this hypothesis in HT disease needs further demonstration. Significantly, PD-1 has been emerging as a potential therapeutic target for ameliorating autoimmune disease [46]; the same prospect could be expected in thyroid aspects.

## 5. Conclusion

In this study, our data indicated that HT patients exhibit low percentage of classical Tregs and aTregs, and these cells had dynamic changes in the progression of HT. We also found decreased expression of Helios on aTreg cells in HT, whereas PD-1 expression on sTreg and memory Teffect cells was peripherally expanded. More importantly, Tregs and Helios had various associations with serum levels of thyroid-specific autoantibodies, as well as thyroid function in patients with HT. These findings suggest the change of Tregs number and function might be involved in the immunopathogenesis of HT. However, the relatively small sample size is a limitation to address this issue. More samples and further work is necessary to dissect the exact relationship between Tregs and the pathogenesis of HT. Furthermore, it is speculated that Tregs may serve as a novel therapeutic target, and Treg-based intervention is supposed to be implicated in future therapeutics for HT patients.

## Figures and Tables

**Table 1:** Clinical and demographic features of the participants.

	HC	eHT	sHT	oHT	HT
Number	20	20	12	10	42
Sex (F/M)	15/5	19/1	9/3	9/1	37/5
Age (years)	38.85 ± 8.07	41.4 ± 13.55	40.9 ± 11.89	37.2 ± 14.59	40.29 ± 13.15
FT3 (pmol/L)	4.48 (4.28 -5.01)	4.35 (3.94 - 4.68)	4.42 (4.18 - 5.1)	3.34 (2.52 - 4.26)^a^	4.21 (3.7 - 4.72)
FT4 (pmol/L)	15.5 (14.27-16.65)	15.28 (13.97-18.01)	14.92 (13.5-16.35)	9.67^a b c^ (5.11-10.86)	14.46 (12.15-16.54)
TSH (mIU/L)	2.25 (1.82-2.84)	2.11 (1.59-3.42)	5.82 (4.68-6.7)^a b^	36.9 (23.68->100)^a b^	4.45 (2.11-11.02)
TPOAb (IU/ml)	13.0 (6.5 -22.75)	173.2^a^ (69.97 -235.77)	248.45^a^ (172.88-562.6)	473.5^a b^ (306.25- >600)	238.55 (160.9 -479.45)
TgAb (IU/ml)	32.21 (20.25 -51.08)	534.35^a^ (310.15 -623.48)	402.05^a^ (146.78-549.7)	1053.3^a^ (797.1 -1835)	545.75 (345.78 -886.7)

Data was expressed as mean±SD or median (25th-75th percentile) on the basis of the distribution. *HC*: healthy controls; *HT*: Hashimoto thyroiditis; *eHT*: euthyroidism; *sHT*: subclinical hypothyroidism; *oHT*: clinical hypothyroidism;* F*: female; *M*: male; *FT3*: free triiodothyronine; *FT4*: free thyroxine; *TSH*: thyroid stimulating hormone; *TPOAb*: antithyroid peroxidase antibodies; *TgAb*: antithyroglobulin antibodies.

^a^ *P *< 0.05, comparison with health controls.

^b^ *P *< 0.05, comparison with eHT group.

^c^ *P *< 0.05, comparison with sHT group.

## Data Availability

The data used to support the findings of this study are available from the corresponding author upon request.

## Abbreviations

HT: Hashimoto thyroiditis
HC: Healthy controls
AITD: Autoimmune thyroid disease
Tregs: Regulatory T cells
aTregs: Activated Treg cells
rTregs: Resting Tregs cells
sTregs: Secreting Treg cells
TPOAb: Antithyroperoxidase antibody
TGAb: Antithyroglobulin antibody
TSH: Thyroid-stimulating hormone
eHT: Euthyroidism
sHT: Subclinical hypothyroidism
oHT: Overt hypothyroidism
PBMCs: Peripheral blood mononuclear cells
PBS: Phosphate buffered saline
FCM: Flow cytometry
RA: Rheumatoid arthritis
SLE: Systemic lupus erythematosus
MS: Multiple sclerosis
T1DM: Type 1 diabetes mellitus
SSc: Systemic sclerosis.
